# Comparing different analysis methods for quantifying the MRI amide proton transfer (APT) effect in hyperacute stroke patients

**DOI:** 10.1002/nbm.3147

**Published:** 2014-06-10

**Authors:** Y. K. Tee, G. W. J. Harston, N. Blockley, Thomas W. Okell, J. Levman, F. Sheerin, M. Cellerini, P. Jezzard, J. Kennedy, S. J. Payne, M. A. Chappell

**Affiliations:** ^1^ Institute of Biomedical Engineering, Department of Engineering ScienceUniversity of OxfordOxfordUK; ^2^ Centre for Doctoral Training in Healthcare InnovationUniversity of OxfordOxfordUK; ^3^ Acute Stroke ProgrammeRadcliffe Department of MedicineOxfordUK; ^4^ Oxford Centre of Functional MRI of the Brain, Nuffield Department of Clinical NeurosciencesUniversity of OxfordOxfordUK; ^5^ Department of NeuroradiologyOxford University Hospitals NHS TrustOxfordUK

**Keywords:** Amide proton transfer (APT) imaging, chemical exchange saturation transfer (CEST) imaging, magnetization transfer (MT) imaging, pH, MRI, stroke

## Abstract

Amide proton transfer (APT) imaging is a pH mapping method based on the chemical exchange saturation transfer phenomenon that has potential for penumbra identification following stroke. The majority of the literature thus far has focused on generating pH‐weighted contrast using magnetization transfer ratio asymmetry analysis instead of quantitative pH mapping. In this study, the widely used asymmetry analysis and a model‐based analysis were both assessed on APT data collected from healthy subjects (*n* = 2) and hyperacute stroke patients (*n* = 6, median imaging time after onset = 2 hours 59 minutes). It was found that the model‐based approach was able to quantify the APT effect with the lowest variation in grey and white matter (≤ 13.8 %) and the smallest average contrast between these two tissue types (3.48 %) in the healthy volunteers. The model‐based approach also performed quantitatively better than the other measures in the hyperacute stroke patient APT data, where the quantified APT effect in the infarct core was consistently lower than in the contralateral normal appearing tissue for all the patients recruited, with the group average of the quantified APT effect being 1.5 ± 0.3 % (infarct core) and 1.9 ± 0.4 % (contralateral). Based on the fitted parameters from the model‐based analysis and a previously published pH and amide proton exchange rate relationship, quantitative pH maps for hyperacute stroke patients were generated, for the first time, using APT imaging. © 2014 The Authors. *NMR in Biomedicine* published by John Wiley & Sons, Ltd.

**Abbreviations used**
APT: amide proton transfer
APTR: APT ratio
CERT: chemical exchange rotation transfer
CNR: contrast‐to‐noise ratio
CSF: cerebrospinal fluid
CV: coefficient of variation
DWI: diffusion‐weighted imaging
FAST: FMRIB's Automated Segmentation Tool
FLIRT: FMRIB's Linear Image Registration Tool
GM: grey matter
MT: magnetisation transfer
MTC: conventional magnetisation transfer contrast
MTR_asym_: magnetisation transfer ratio asymmetry
MTR_asym_comp_: magnetisation transfer ratio asymmetry complementary
NOE: nuclear Overhauser enhancement
ROI: region of interest
WM: white matter

## INTRODUCTION

The goal of defining the ischaemic penumbra in human acute ischaemic stroke using imaging that can be widely used in clinical practice has proved elusive. Much effort has focused on the imaging of regional cerebral blood flow, but this has not reliably identified individuals who might benefit from targeted intervention, such as endovascular treatment 1. The original description of the penumbra suggested that imaging should also capture a measure of tissue metabolism 2. The measurement of intracellular pH is an attractive marker for understanding tissue metabolic stress, given that tissue pH is maintained until immediately prior to irreversible cerebral infarction 2, 3. Therefore, pH‐weighted imaging offers the possibility of targeting intervention at a stage at which there may be reversibility of ultimate tissue outcome, although the reliable use of this technique in this population has been challenging 4.

Intracellular pH can be estimated by the quantification of amide proton transfer (APT) using the chemical exchange saturation transfer phenomenon 5, which measures the transfer of protons between the amide groups on metabolites and proteins with water; this process is base‐catalysed in the physiological pH range (pH 6.5–7.5) and hence is pH dependent. The estimation of intracellular pH through APT imaging has been demonstrated in preclinical models 5, 6. A previous study has shown that the quantified APT effect in the preclinical models is highly correlated with the final infarct tissue area defined using *T*_2_ hyperintensity at 24 h 7, suggesting that thresholds may exist to define tissue outcome following ischaemic stroke. It is generally agreed that the APT effect decreases in the infarcted and salvageable tissue during the hyperacute period, owing to pH reduction 5, 8, 9.

APT imaging in patients with ischaemic stroke has been reported 4, 10. Despite the potential of APT imaging to measure pH, most of the literature so far has focused on the generation of pH‐weighted contrast rather than quantitative pH maps. The most commonly used metric to measure pH‐weighted saturation transfer images is magnetisation transfer ratio asymmetry (MTR_asym_) at offsets of ±3.5 ppm relative to water 11, 12, 13, 14. However, this metric is dependent on experimental parameters, such as power and duration of saturation 15, and contaminated by various other effects 12, 14, 16, such as direct water saturation, conventional magnetisation transfer contrast (MTC), other metabolites which resonate near amide protons and nuclear Overhauser enhancement (NOE), which span across a range of negative frequency offsets from water. Furthermore, NOE has recently been shown to be pH sensitive under low‐power saturation 17, making the use of the signal measured at −3.5 ppm less suitable as a reference for APT quantification.

The purpose of this study was to compare the sensitivity of quantitative Bayesian model‐based analysis 18 and conventional approaches, such as MTR_asym_, to APT data_._ Two groups of subjects (healthy volunteers and hyperacute stroke patients presenting within 6 h of onset) were scanned to establish the most suitable APT quantification method. This was defined as the analysis method that produced the smallest coefficient of variation (CV) in healthy tissue and the greatest contrast variation between infarct core and normal tissue in the acute stroke patients. In addition, the absolute intracellular pH was calculated from APT measurements to test the stability of the experimentally derived models in clinical patients.

## THEORY

### Asymmetry analysis

In an APT imaging experiment, saturation is usually performed across a range of frequency offsets and the measured saturated signal plotted against offset is commonly referred to as the *z*‐spectrum. The APT effect is often calculated, following the practice in magnetisation transfer (MT) imaging, using MTR_asym_:

(1) $${\mathrm{MTR}}_{\mathrm{asym}}=\frac{M\left(-\Delta \omega \right)-M\left(+\Delta \omega \right)}{M_{0}}\;$$

where *M*(±Δ*ω*) refers to the measured saturated signal at ±3.5 ppm and *M*_0_ is the measured unsaturated signal. This analysis inherently assumes that all the non‐APT effects, such as direct saturation, and MTC effects are symmetric about the water centre frequency; thus, the pure APT effect can be obtained by taking the difference between the signals measured at positive and negative offsets.

Various other forms of asymmetry measure, based on the concept of Equation (1), have been studied using phantoms, paramagnetic chemical exchange saturation transfer agents and animal models, and it has been found that a complementary approach, denoted as ST^com^, where ST stands for saturation transfer, is more suitable for *in vivo* applications 19. For consistency in this work, ST^com^ is renamed as MTR_asym_comp_, and can be calculated using the following equation:

(2) $${\mathrm{MTR}}_{\mathrm{asym}\_\mathrm{comp}}=\frac{M\left(-\Delta \omega \right)-M\left(+\Delta \omega \right)}{M_{0}-M\left(-\Delta \omega \right)}\;$$

Recent studies have shown that the symmetric assumption does not hold, especially *in vivo*, because of inherent asymmetries in MTC or contributions from NOE 12, 16, 17, 20. Hence, the quantification of the APT effect using MTR_asym_ contains not only the APT effect (APT ratio, APTR), but also contributions from other transfer of magnetisation effects (MTR'_asym_):

(3) $${\mathrm{MTR}}_{\mathrm{asym}}=\mathrm{MT}R_{\mathrm{asym}}^{\prime }+\mathrm{APTR}\;$$

As a result of the contributions from MTR'_asym_, quantification of the APT effect using the above metric often results in a negative amplitude when a low saturation power is used, which would not be expected from a symmetric *z*‐spectrum 18, 21; thus, these metrics are usually regarded as producing APT‐ or pH‐weighted images. Although the complementary approach [Equation (2)] is claimed to perform better than the conventional metric in a biological environment, it still suffers from the same confounding factors in the negative frequency offsets.

### Model‐based analysis

In order to quantify the pure APT effect, a model‐based approach has been proposed 18, 22, 23, 24, in which the full modified Bloch equations 25, 26 are fitted to the measured data, including pools for water, amide and asymmetric MTC effects. Using the fitted parameters from the model‐based analysis, a further quantitative metric can be calculated, denoted as APTR*. This measure uses the parameters from the model fitting to generate an ideal two‐pool, water and amide (w + a), *z*‐spectrum and to compare it with an ideal one‐pool model of water (w) to obtain the pure APT effect 18, 24:

(4) $${\mathrm{APTR}}^{*}=\frac{S_{w}\left(\Delta \omega \right)-S_{w+a}\left(\Delta \omega \right)}{M_{0}}\;$$

where *S*(Δ*ω*) is the simulated signal at 3.5 ppm using the fitted parameters and the subscripts ‘w’ and ‘w + a’ refer to fitted parameters from the water pool and both the water and amide pools, respectively. Although a greater number of pools can be used to perform the model fitting as required, and thus to correct for asymmetries in the *z*‐spectrum, only the fitted model parameters of water and amide pools are used here to calculate APTR*. In principle, APTR* provides a single quantitative measure of the APT effect (combining both amide proton exchange rate and concentration contributions) without *B*_0_ inhomogeneity, direct saturation, MTC and NOE influences, as the latter effects (MTC and NOE) can be accounted for by grouping them as a separate pool in the model fitting 18, 27.

## MATERIALS AND METHODS

### Subject recruitment

Patients with acute stroke were recruited following informed consent or agreement from a representative according to a research protocol agreed by the UK National Research Ethics Service Committee South Central (ref: 12/SC/0292). The data presented in this study were acquired from the first six patients recruited with lesions greater than 2 cm in diameter on diffusion‐weighted imaging (DWI) and without significant artefact. The median age of the studied patients with acute stroke was 83 years. The median onset of symptoms to research MRI scan was 2 h 59 min (range from 1 h 43 min to 5 h 46 min). Three patients received intravenous tissue plasminogen activator during the MRI scan. Two healthy male volunteers (28 and 36 years) were also scanned to act as controls.

### MRI experiment

All MRI scans were performed using a 3 T Siemens Verio scanner (Siemens Medical Solutions, Erlangen, Germany) in the Oxford Acute Vascular Imaging Centre located at the John Radcliffe Hospital, Oxford, UK. The following MRI sequences were acquired from the patients with hyperacute stroke: *T*_1_ structural image with a spatial resolution of 1.781 × 1.781 × 1 mm^3^; DWI with three directions (*b* = 0 and 1000 s/mm^2^); and single‐slice APT imaging with a spatial resolution of 3.4 × 3.4 × 5 mm^3^, where the plane was selected by an attending clinician based on the lesion seen on the DWI scan. The APT saturation was performed with 50 Gaussian pulses; each pulse had a flip angle of 184° and duration of 20 ms with 20 ms spacing, to achieve an equivalent continuous saturation *B*_1_ value of 0.55 μT (average power) for 2 s. Crusher gradients were applied between pulses to spoil the residual transverse magnetisation. A spin‐echo echo planar imaging readout (TR = 5 s, TE = 23 ms, matrix 64x64 and 6/8 partial Fourier) was performed after all the Gaussian pulses had been applied. Data were acquired for a range of saturation frequency offsets; the offsets were evenly distributed from −4.5 to 4.5 ppm with a 0.3 ppm interval for Patients 1 and 2, and the unsaturated data were acquired with no radiofrequency saturation; the rest had offsets at −50, −30, −4.1, −3.8, −3.5, −3.2, −2.9, −0.9, −0.6, −0.3, 0, 0.3, 0.6, 0.9, 2.9, 3.1, 3.2, 3.3, 3.4, 3.4, 3.5, 3.5, 3.6, 3.6, 3.7, 3.8, 3.9, 4.1, 30, 50 and ±300 ppm (treated as unsaturated images). Both sampling schedules acquired 32 APT images in approximately 3 min. For the healthy subjects, only the *T*_1_ structural and APT scans were acquired.

### Data processing and analysis

The Brain Extraction Tool in the FSL package 28 was used to remove the skull and non‐brain areas in all the collected data. All the imaging modalities were transferred to the *T*_1_ space using FMRIB's Linear Image Registration Tool (FLIRT) in the FSL package 29. Rigid body registration with six degrees of freedom and correlation ratio cost function was applied to register each imaging modality to *T*_1_, except for the single‐slice APT data.

For the APT data, the different frequency offset brain images were first aligned to the unsaturated APT brain image using two‐dimensional rigid body registration with three degrees of freedom. A three‐pool model consisting of water (w), amide (a) and conventional magnetisation transfer contrast + nuclear Overhauser enhancement (MTC + NOE), following ref. 18, was then fitted pixelwise to the motion‐corrected *z*‐spectrum using a Bayesian algorithm 30 (www.fmrib.ox.ac.uk/fsl/baycest), with the prior values of each parameter given in Table 1 and treating the pulsed saturation as its continuous approximation using average power 23.

**Table 1:** Model parameters with prior values expressed as the mean and standard deviation of a normal distribution, modified from ref. 18

	Water pool		Amide pool		MTC + NOE pool	
Parameter^a^	Mean	SD	Mean	SD	Mean	SD
*M*_0_	0	10^6^	–	–	–	–
*M*_0_ *^i^*/*M*_0_^w^	–	–	0.09/112^b^	0.02/112^b^	0	0.01
ln(*k*_*i*–w_)	–	–	3.0	1.0^c^	3.4	1.0
*T*_1_ (s)	1.3	0.15	0.77	0.15	1.0	0.15
*T*_2_ (ms)	70	14	10	2	0.2	0.04
*ω* (ppm)	0	0.1	3.5	0.1	−2.41	0.1

a *M*_0_, initial magnetisation; *k*, exchange rate; *T*_1_, longitudinal relaxation time; *T*_2_, transverse relaxation time; *ω*, chemical shift of each pool with respect to water; *i*, amide or conventional magnetisation transfer contrast + nuclear Overhauser enhancement (MTC + NOE) pool.

b The mean of the *in vivo* amide concentration was set as 90 mm so that a standard deviation of 20 mm would include the reported values in the literature, 72 mm 5 and 100 ± 8 mm 20, where 112 m is the concentration of water protons. In the APTR* *versus* pH simulations, the amide concentration was assumed to be 100 mm instead of 72 mm because the latter was estimated without corrections to various possible contaminations.

c The mean of the amide proton exchange rate was set to be 20 Hz and a natural logarithm was used to make the model parameter closer to linear to facilitate convergence of the algorithm.

The shift in the *z*‐spectrum caused by *B*_0_ inhomogeneity was corrected pixelwise using the fitted water centre frequency from the model fitting. MTR_asym_ was obtained by finding the area under the curve from 3.3 to 3.7 ppm according to Equation (1) using the shift‐corrected *z*‐spectrum at each pixel; likewise MTR_asym_comp_ was calculated following Equation (2), where *M*_0_ became the unsaturated signal multiplied by the frequency range considered (3.7 ‐ 3.3 ppm = 0.4 ppm).

Previously, a relationship between the base‐catalysed amide proton exchange rate,  *k*_a − w_, and intracellular pH has been calibrated using both phosphorus and water exchange spectroscopy 5, where:

(5) $$\mathrm{pH}=6.4+\log_{10}\left(\frac{k_{a-w}}{5.57}\right).$$

For the proposed model‐based approach, an idealised APTR* *versus* pH relationship could also be formed using the relationship above and simulations. By assuming water and amide proton concentrations of 112 m and 100 mm 20, respectively, and the remaining parameters in the two‐pool model (water and amide) having the mean values in Table 1, saturated by *B*_1_ = 0.55 μT and a saturation time of 2 s (to match the experiment), a range of idealised APTR* *versus* different pH values [calculated from Equation (5) by varying the amide proton exchange rates] could be simulated, and a relationship between them could then be formed.

For the collected APT data, the idealised APTR* could be calculated according to Equation (4) using only three parameters from the model fitting: (1) the fitted unsaturated signal, *M*_0_^w^; (2) the fitted amide proton exchange rate, *k*_a–w_; and (3) the fitted amide proton signal, *M*_0_^a^. As the variations in the remaining parameters in the ideal one‐ and two‐pool models should have been accounted for by the fitting algorithm, they were assumed to have values from the simulations used to generate the idealised APTR* *versus* pH relationship. Both *k*_a–w_ and *M*_0_^a^ were used to calculate the idealised APTR* instead of *k*_a–w_, which is related to pH, because both of these parameters will affect the *z*‐spectrum at the amide resonance, and it has been proved difficult to separate their effects in model fitting using a single saturation power 18, 24. *M*_0_^w^ was also included to normalise the measured saturated signal. The idealised APTR* is simply referred to as APTR* hereafter.

The quantified APT effects using the different metrics (MTR_asym_, MTR_asym_comp_ and APTR*) were transformed to the *T*_1_ image space using FLIRT. The *T*_1_ structural data were segmented using FMRIB's Automated Segmentation Tool (FAST) 31 into cerebrospinal fluid (CSF), grey and white matter (GM and WM).

### Healthy subjects

Two criteria were used to assess the efficacy of the different quantification methods in the healthy control: (1) contrast between GM and WM in the brain, *C* = (*M*_GM_–*M*_WM_)/(*M*_GM_ + *M*_WM_), where *M* is the average signal of the studied metrics in a region of interest (ROI); and (2) CV to study the precision, defined as the ratio of the standard deviation to the mean of the respective quantification method, in both GM and WM. The most suitable APT quantification method for a healthy brain should produce the smallest CV in the GM and WM, and have minimum contrast between these ROIs. In order to minimise partial volume effects and contamination from non‐brain areas, such as CSF, the GM and WM masks were created using an 80% threshold on the GM and WM partial volume estimates generated by FAST.

### Stroke patients

The DWI data (*b* = 1000 s/mm^2^) were used to define the infarct core, where an infarct core mask for each patient and a mirror mask of normal tissue in the contralateral hemisphere were manually drawn by a clinician. The average *z*‐spectra within the infarct core and contralateral ROIs were plotted to study their differences. The coefficient of determination, *R* ^2^, was calculated to assess the goodness of fit for the model‐based analysis in each ROI. Two‐tailed unpaired *t*‐tests were performed comparing the APT effect between infarct core and the contralateral region using the different approaches to examine which metric was more suitable to distinguish the two tissue types. The *t*‐tests were performed twice for MTR_asym_ and MTR_asym_comp_; once directly on the calculated values (unbounded) and once within a specified range (bounded): −0.05 < MTR_asym_ < 0; −0.5 < MTR_asym_comp_ < 0. This was done to minimise the influence of measurement noise and artefacts, such as imperfect fat suppression.

The contrast‐to‐noise ratio (CNR), defined as CNR = *C*/CV_Contralateral_, where *C* is the contrast between the infarct core and contralateral normal‐appearing tissue and CV_Contralateral_ refers to CV in the contralateral tissue, was calculated to assess which APT quantification method was most suitable for clinical stroke imaging.

## RESULTS

**Figure 1:**
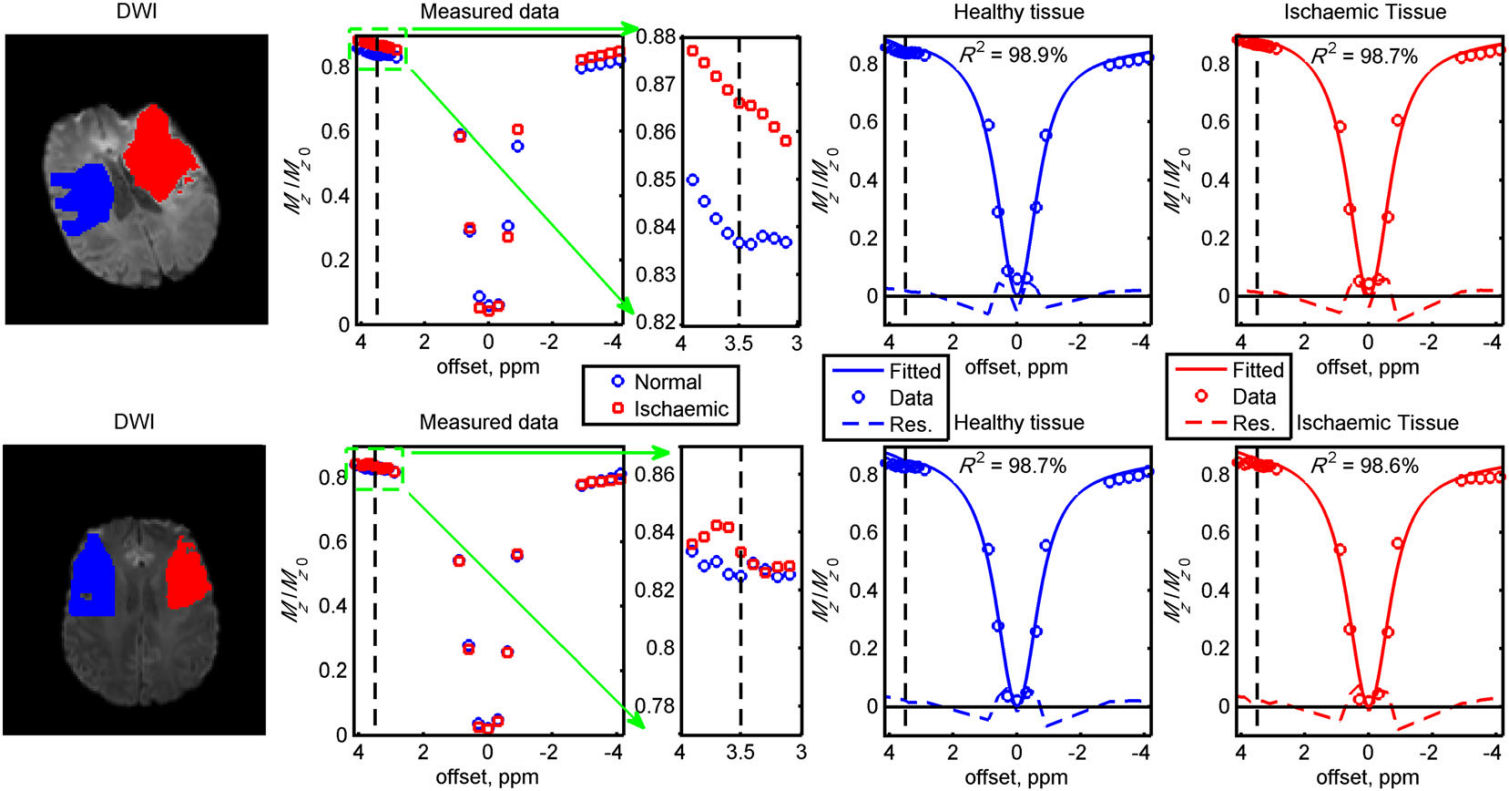
Measured z‐spectra after *B*_0_ correction and model fits in the different regions of interest of Patients 3 (top row) and 5 (bottom row). The red areas indicate the infarct core [hyperintensity on diffusion‐weighted imaging (DWI) data (*b* = 1000 s/mm^2^) acquired during admission] and the blue regions show the contralateral normal‐appearing tissue. Very good fits were obtained in the regions of interest with the coefficient of determinant *R* ^2^ ≥ 98.6%. The vertical black dashed lines represent the chemical shift of amide protons and the blue/red dashed lines underneath the fits and measured data are the residuals.

The representative z‐spectra and model‐based fits in the infarct core (hyperintensity on the DWI data acquired during admission) and the contralateral normal‐appearing tissue of Patients 3 and 5 are plotted in Fig. 1. From the measured data, all the z‐spectra in the infarct core were found to have a higher magnitude of magnetisation ratio (smaller saturation effect) at the chemical shift of amide protons when compared with the contralateral ROI (Figs 1 and S1). Very good fits were obtained using the three‐pool model, where *R* ^2^ ≥ 98.6% in the ischaemic and contralateral ROIs. The main discrepancy between the fits and measured data was found to be around the water centre frequency.

**Figure 2:**
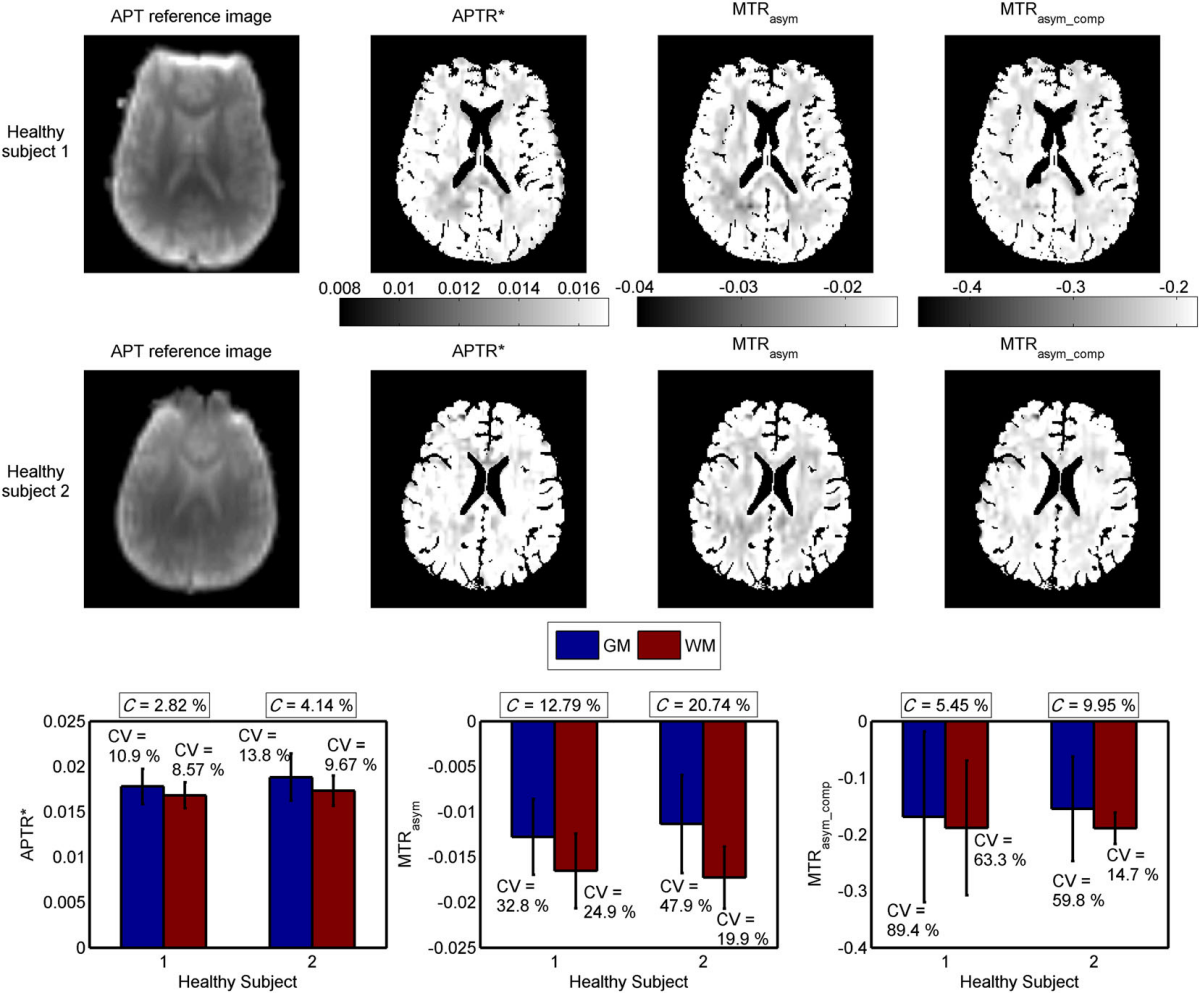
Amide proton transfer (APT) reference images and calculated maps of APTR*, MTR_asym_ and MTR_asym_comp_ of healthy volunteers. Tissue masks were used to remove the non‐tissue areas, such as cerebrospinal fluid (CSF); thus, the scale bar does not reflect the quantified APT effect in these areas. The bar graphs and error bars are the means and standard deviations, respectively, of each metric in the grey matter (GM) and white matter (WM). CV stands for coefficient of variation and *C* refers to the contrast between GM and WM.

The quantified APT effects in the healthy volunteers using the different metrics are shown in Fig. 2. There were some intra‐ and inter‐subject variations in the quantified APT effect using the different methods in GM and WM, but no obvious low signal areas were observed. The mean, standard deviation and CV of GM and WM, together with the contrast from each metric, are presented in the bottom row of Fig. 2. The APT effect was found to be negative when asymmetry measures (MTR_asym_ and MTR_asym_comp_) were used, with the APT effect in WM more negative than in GM. When the APT effect was quantified using APTR*, positive differences were obtained; WM had a consistently smaller APTR* magnitude than GM. APTR* was found to have the smallest CV in GM and WM (≤13.8%), and also the smallest average contrast between these tissue types (*C*_avg_ = 3.48%). MTR_asym_ had the highest contrast between GM and WM, but a smaller CV than MTR_asym_comp_, in each region.

**Figure 3:**
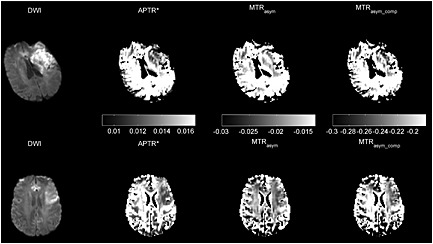
Diffusion‐weighted imaging (DWI) (*b* = 1000 s/mm^2^) and processed amide proton transfer (APT) results (APTR*, MTR_asym_ and MTR_asym_comp_) from representative patients: top row, Patient 3; bottom row, Patient 5. Tissue masks were used to remove the non‐tissue areas, such as cerebrospinal fluid (CSF); thus, the scale bar does not reflect the quantified APT effect in these areas.

Figure 3 shows the DWI data and the calculated APT effect using MTR_asym_, MTR_asym_comp_ and APTR* for representative Patients 3 and 5. In Patient 3 (top row of Fig. 3), APTR* and MTR_asym_comp_ showed a low signal area in the infarct core identified from the area of hyperintensity in the DWI image (*b* = 1000 s/mm^2^). In the MTR_asym_ image of this patient, the difference between ischaemic and healthy tissue was less obvious when compared with the other two metrics. Results from Patient 5 are shown in the bottom row of Fig. 3; it was observed that all three metrics showed a different degree of signal reduction in the infarct core. The low signal areas of APTR* and MTR_asym_comp_ were slightly larger than the area of hyperintensity in the DWI image.

**Figure 4:**
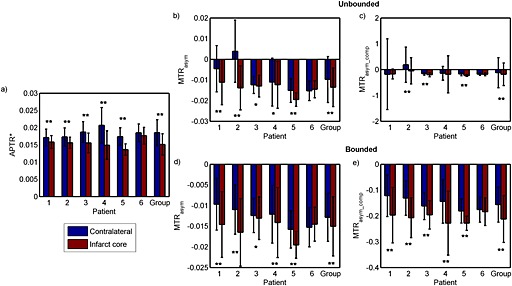
Results of unpaired *t*‐tests between the infarct core and contralateral normal‐appearing tissue region of APTR*, MTR_asym_ and MTR_asym_comp_, where the significant differences are labelled as: *p < 0.05; **p < 0.001. The ‘Group’ label represents the average results of all patients. The bar graphs and error bars refer to the means and standard deviations of each metric, respectively. Bounded results (d and e) include only MTR_asym_ within 0 and −0.05, and MTR_asym_comp_ within −0.5 and 0, whereas the unbounded results (b and c) include all the calculated values.

The means and standard deviations of each metric in the infarct core and contralateral normal‐appearing tissue area of each patient are shown in Fig. 4, together with the results of the *t*‐tests. Significant differences between APTR* in the infarct core and contralateral tissue were found for all patients (*p* < 0.001, labelled ‘**’ in Fig. 4a), except Patient 6. The calculated APTR* was consistently lower in the infarct core than in the contralateral normal‐appearing tissue, with the group means and standard deviations equalling 0.015 ± 0.0031 and 0.019 ± 0.0037, respectively. When the unbounded MTR_asym_ and MTR_asym_comp_ in these regions were compared using *t*‐tests (Fig. 4b, c), some of the results showed significant differences, but large standard deviations were observed, for example, MTR_asym_comp_ in Patients 1 and 2. When the bounded MTR_asym_ and MTR_asym_comp_ between the infarct core and contralateral normal‐appearing tissue were analysed, significant differences between these two regions were found for all patients (labelled ‘*’ for *p* < 0.05 and ‘**’ for *p* < 0.001 in Fig. 4d, e), except for MTR_asym_ and MTR_asym_comp_ in Patient 6. The quantified APT effect using the asymmetry measures (MTR_asym_ and MTR_asym_comp_) showed a more negative signal in the infarct core when compared with the contralateral tissue in all cases, except for MTR_asym_ in Patient 6. The group means and standard deviations of MTR_asym_ were −0.013 ± 0.0058 in the contralateral region and −0.015 ± 0.0072 in the infarct core. For MTR_asym_comp_, the group results were −0.157 ± 0.068 in the contralateral region and −0.212 ± 0.091 in the infarct core. There were inter‐subject variations for all the metrics investigated in either of the ROIs.

Table 2 shows the contrast between the infarct core and contralateral normal‐appearing tissue and the calculated CV in the contralateral tissue for all patients; for the asymmetry analyses (MTR_asym_ and MTR_asym_comp_), only the bounded results are included. Although the overall contrast of APTR* for all the patients was not as high as for the other two metrics, it was able to quantify the contralateral APT effect with smaller variation. Thus, APTR* was able to produce the highest CNR, followed by MTR_asym_comp_ and MTR_asym_.

**Table 2:** Contrast (*C*) between infarct core and contralateral normal‐appearing tissue, and coefficient of variation (CV) in the contralateral tissue of APTR*, MTR_asym_ and MTR_asym_comp_ of each patient. All the results are expressed in absolute percentage values (%); only bounded MTR_asym_ and MTR_asym_comp_ are included

Patient	*C*	CV_Contralateral_	CNR = *C*/CV_Contralateral_
APTR*			
1	4.01	14.2	28.24
2	5.15	15.2	33.88
3	9.31	16.1	57.83
4	16.16	25.0	64.64
5	12.09	14.7	82.24
6	2.27	13.9	16.33
		Average	47.19
MTR_asym_			
1	20.26	64.6	31.36
2	20.00	54.1	36.97
3	2.54	33.4	7.60
4	7.74	57.6	13.44
5	10.61	28.6	37.10
6	2.53	31.0	8.16
		Average	22.44
MTR_asym_comp_			
1	23.50	65.9	35.66
2	22.48	50.4	44.60
3	9.46	32.5	29.11
4	22.62	58.4	38.73
5	11.58	25.9	44.71
6	2.30	27.6	8.33
		Average	33.52

CNR, contrast‐to‐noise ratio.

**Figure 5:**
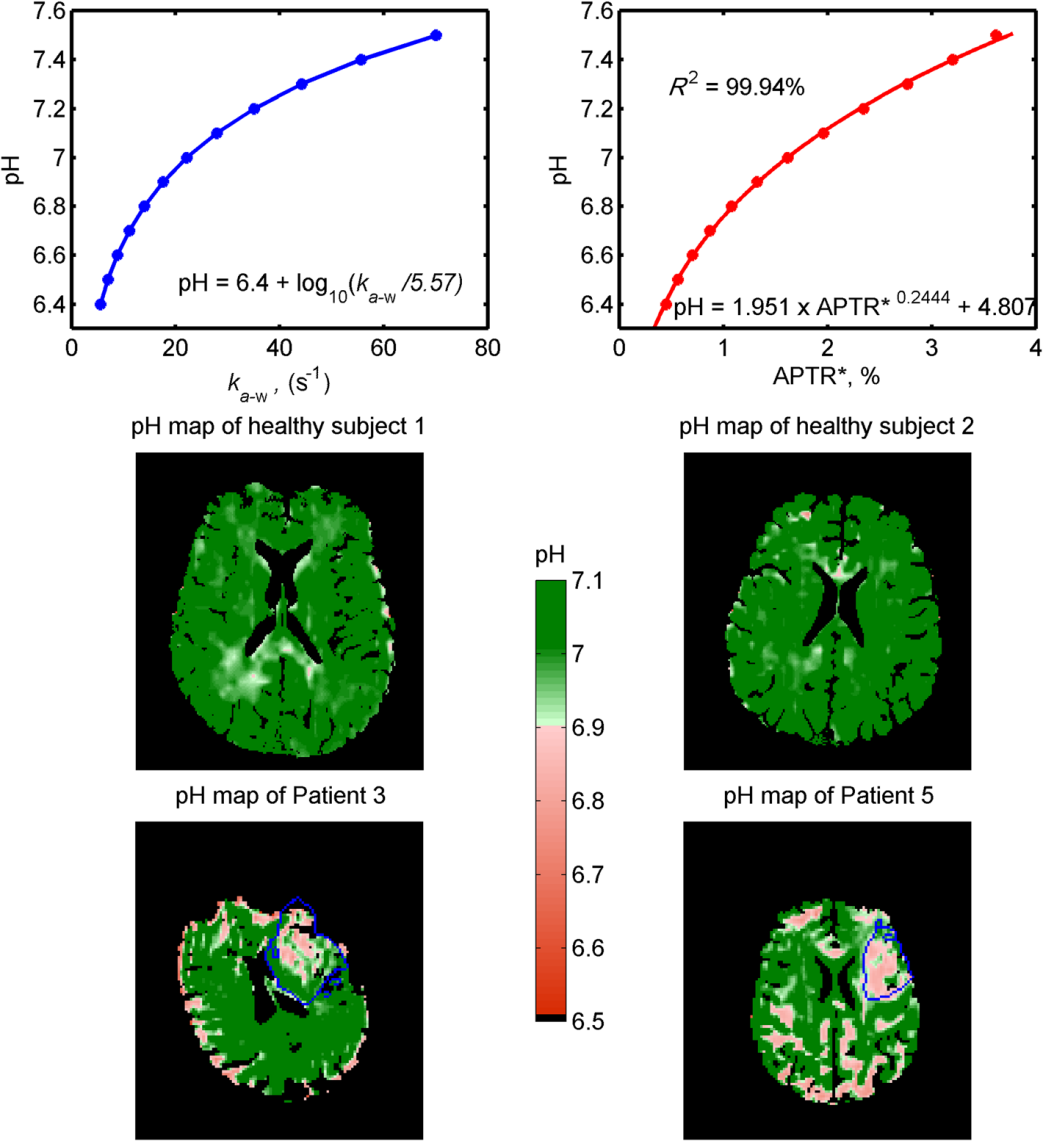
Top left: a logarithmic relationship between pH and amide proton exchange rate proposed by ref. 5. Top right: the pH *versus* APTR* relationship formed by simulations; very good fits were obtained (*R* ^2^ = 99.94%) for pH 6.4–7.5. The middle and bottom rows show the quantitative pH maps of healthy subjects and patients illustrated in Figs 2 and 3, respectively, generated using the pH and APTR* relationships found. The pH scale is set based on the logarithmic relationship study 5, where pH 7.11 ± 0.13 is normal (green) and below pH 6.9 is ischaemic (pink to red), which effectively thresholds the results. The blue lines in the patient images represent the area of the infarct core defined on the basis of the diffusion‐weighted imaging (DWI) data. Tissue masks were used to remove the non‐tissue areas, such as cerebrospinal fluid (CSF); thus, the colour scale does not reflect the quantified amide proton transfer (APT) effect in these areas.

Previously, a logarithmic relationship between the intracellular pH and amide proton exchange rate has been derived using both phosphorus and water exchange spectroscopy 5 (Fig. 5, top left). Using this logarithmic relationship and assuming that the model parameters in water and amide pools had the mean values in Table 1, a further relationship between pH and APTR* was formed, where pH = 1.951 × (APTR*)^0.2444^ + 4.807 (Fig. 5, top right). Based on the pH and APTR* relationship formed, quantitative pH maps of the healthy volunteers and patients were produced, as shown in Fig. 5. Relatively homogeneous pH maps were obtained for the healthy subjects, with tissue having pH values of 7.04 ± 0.07. The pH colour bar was set according to ref. 5, where 7.11 ± 0.13 is normal (green) and below 6.9 is ischaemic (pink to red), which effectively thresholds the results. In representative patient cases, lower pH values (Fig. 5, blue lined areas, pH = 6.94 ± 0.14) were noticeable in the infarct core, as identified by a clinician based on hyperintensity on the DWI data at admission. Lower pH values were also observed around the edge of the brain and tissue areas proximal to CSF in patient data, as well as in healthy subjects, but to a much smaller extent when compared with the former.

## DISCUSSION

This study examined different ways of quantifying the APT effect on data acquired in patients with hyperacute stroke: MTR_asym_, MTR_asym_comp_ and APTR*. From the results, APTR* was able to generate the smallest contrast between GM and WM, and CV in the healthy subjects, which is desirable. All the quantification methods showed a more elevated APT effect in GM than WM (Fig. 2), which has been observed previously by others 18, 21, 32, 33. This most probably relates to differences in labile proton concentrations and is something that none of the methods considered here can truly control for.

When APTR* was applied to quantify the APT effect in the data from patients with hyperacute stroke, the quantified effects were consistently lower in the infarct core relative to the contralateral normal‐appearing tissue for all patients, and statistically significant differences at a 5% significance level were found between these tissue regions for all patients, except Patient 6. Without setting a proper bound for the statistical analyses, MTR_asym_ and MTR_asym_comp_ were found to have large variations within the ROIs. These asymmetry measures appear to be more susceptible than the model‐based analysis to noise and artefacts. MTR_asym_ and MTR_asym_comp_ were found to be mostly negative, unlike APTR*, this being observed previously 18, 32 when relatively low RF saturation powers are employed. Thus, any pH estimation based on the asymmetry measures would be more strongly influenced by the aliphatic signal (negative offsets), conflicting with its intended use as a metric to describe the APT effect. Nevertheless, the asymmetry analysis method did demonstrate the ability to identify apparent APT effects. Since they can be calculated more quickly than the model‐based approach, they remain suitable as a first approximation and as a means of generating non‐quantitative contrast images.

APT experiments are commonly performed using a higher saturation power than that used in this study to maximise the contrast and to generate a positive difference when asymmetry analysis is used to quantify the effect. However, a high power contributes to large MTC and spillover effects 33, making the use of asymmetry analysis less specific to the APT effect alone. Based on efficiency theory 12, 21, only a small power is required to fully saturate the protons that are in slow exchange with the water protons, such as amide, at approximately 28 Hz 5. Therefore, the APT experiments in this study were performed using a small irradiation power to minimise the contamination from other transfer of magnetisation effects. However, the difference in the irradiation power used may have influenced the efficacy of the asymmetry measures here.

In order to speed up the model‐based analysis, the average power of the Gaussian pulses was used instead of a discretisation method which segments each pulse into *n* intervals; the final magnetisation is achieved by propagating the calculation through all the intervals, with the calculated signals at one segment used as the initial conditions for the following one. As a result of this simplification, the model fitting was not able to fit the signal well around the water centre frequency (Fig. 1) because of the difference in the bandwidth between the irradiation scheme used and that assumed in the model; a continuous irradiation scheme is able to saturate the pool more efficiently, leading to narrower off‐resonance excitation when compared with the pulsed saturation used in this study. One of the main reasons for using the continuous approximation, even though it affects the model fitting, is to make the quantification method more clinically relevant in term of processing time. The APTR* and pH information can be generated in less than 10 min on a normal PC with eight cores of processors by processing the APT data in parallel. The fitting accuracy around the water centre frequency can be improved using the computationally expensive discretisation method 23. Nevertheless, the differences between the important fitted parameters, such as the amide proton exchange rate, using the continuous approximation and discretisation method, for protons with a slow exchange rate, should be small; thus, this should not significantly affect the APTR* results here 23.

When the average z‐spectra across a ROI in the ischaemic and contralateral tissue of patients were analysed, the APT effect was observed to decrease in the infarct core, as was the signal in the aliphatic range, when compared with the contralateral side, for some of the patients, as shown in Fig. 1. The change at 3.5 ppm has been attributed to acidosis, which leads to a slower amide proton exchange rate and thus a smaller APT effect 5. The post‐stroke effect in the aliphatic range has not been widely reported. A previous study in a preclinical model has suggested that the saturation effect at the negative offsets is pH insensitive 34, whereas the recent literature on phantoms has shown that the effect is pH dependent 17 under low‐power saturation, such as the study presented here. This discrepancy may arise from the different RF irradiation powers used, because NOE has been shown to be power dependent 35. The change at the negative frequency offsets is not surprising because the brain undergoes cascaded and complex physiological adaptation after ischaemic injury. These adaptations have been reported previously by many spectroscopy studies, including noticeable changes in the aliphatic range during severe energy failure 36 and an increase in neutral lipid droplets in the infarcted region following induced stroke 37.

Using the relationship of pH and APTR* found from the simulation, quantitative pH maps were demonstrated in healthy subjects and in patients with hyperacute stroke. When the proposed method was applied to the healthy volunteers, relatively homogeneous pH maps were obtained, which is expected, reflecting the assumption that APT provides a direct measure of pH because the body will try to maintain homeostasis. In patients, lower pH values were observed in the ischaemic area, as shown in Fig. 5. To the best of our knowledge, these are the first quantitative pH maps generated from APT imaging in hyperacute stroke patients. The relationship used here is specific to the saturation scheme employed, although the methodology could be employed to generate a similar relationship for other schemes. Ideally, only the exchange rate constant would be extracted from the model‐based analysis and used to calculate pH, but it remains difficult to separate the effects of exchange rate and proton concentration from a sampled z‐spectra 18, 24.

Although realistic pH maps were generated in this study, artefacts (low pH values) were seen in the non‐ischaemic tissue areas, a problem that was particularly noticeable in the patient cohort. There are several factors that might explain this. First, Equation (5) was derived using phosphorus spectroscopy, which primarily assesses the intracellular pH, and by assuming that phosphate species experience the same intracellular environment as amide protons 5; this remains to be validated. Second, the derived relationship [Equation (5)] has been found to show some discrepancies in relation to an APT study carried out by a different centre 9. The discrepancies have been suggested to be a result of the calibrated MTR_asym_ *versus* pH relationship, which may not be the same at different centres because the imaging technique is not standardised 16 and MTR_asym_ is dependent on the acquisition method. Furthermore, as MTR_asym_ does not contain the APT effect alone, recalibration may be necessary to form a more accurate relationship between pH and APTR*, as the latter can quantify the pure APT effect by removing all the other contaminating effects using the model‐based analysis. Finally, the concentration of amide protons was set to be 100 mm across the brain in the simulations to generate the pH and APTR* relationship. This is probably the main factor contributing to the artefacts seen on the generated pH maps, aside from the motion artefacts, because there is zero or minimal endogenous mobile protein and peptide concentration in non‐tissue areas, such as the CSF. The partial volumes of these low or no amide concentration regions within the tissue area may cause the model to estimate them as having a low APT effect. This effect is likely to be more pronounced in the older patients observed here compared with the younger healthy subjects because of atrophy. The low APT effect caused by partial volume contamination has also been reported by others using MTR_asym_ 10, suggesting that a better post‐processing partial volume correction technique may be required, because the APT slice is normally thicker than the *T*_1_ structural image used for segmentation.

The published literature on the application of APT imaging in patients with stroke to date has a mean onset time of hours 4 to 4.3 days 10 after the onset of ischaemia. Even though some of the patients were scanned in the delay phase (days after onset), the APT effect quantified using MTR_asym_ still showed a lower signal in the ‘ischaemic’ area when compared with the contralateral normal‐appearing tissue. However, the quantified signal may not reflect the pH effect alone, because the initial assumptions regarding the negligible change in water relaxation time and proton concentration may well not hold at this stage of stroke evolution. Oedema formation and inflammation are common secondary phenomena after stroke that can alter the magnetic properties of water. There may also be a change in the amide proton concentration as a result of changes in protein synthesis and degradation, and the loss of cytoplasm, days after onset. In addition, a change in the diffusivity in the ischaemic area could possibly affect the exchange rate between water and amide protons. As a result, various other MRI acquisitions are required to account for all of these variations, such as the methods used in a previous preclinical study 6 in order to quantify the APT effect using asymmetry analysis. However, these extra scans will increase the patient scan time and are thus not practical in the setting of acute stroke, supporting the use of model‐based analysis, which can account for all of these variations.

It should be noted that a number of assumptions were made when calculating APTR* and converting it to quantitative pH information. When performing the model fitting, only three pools were considered; the possible contributions from other labile protons, such as amine and hydroxyl protons, to the APT effect were assumed to be minimal, as a low saturation power was used in this study. In the third pool, it was assumed that MTC and NOE could be represented as a single pool, which has been done previously in the literature 18, 27. However, combining these two transfer mechanisms might oversimplify the *in vivo* environment, and there is a possibility that, with a model‐driven analysis, the combined MT and NOE pool implemented here might be suboptimal, and this could in turn affect the *B*_0_ estimation. One of the possible solutions is to exclude the NOE data and only fit the measured signal around amide and water pools using a three‐pool model, where the third pool models only the MTC effect. There is certainly scope to investigate this issue further, especially if NOE is to be used to assess ischaemic injury.

Based on a previous study 5, we assumed that there was negligible change in the water relaxation parameters during early stroke onset. By doing so, a simple APTR* *versus* pH relationship could be formed; it may be necessary to account more fully for changes in relaxation parameters in ischaemic tissue. Although multiple assumptions were made when quantifying the APT effect using the proposed model‐based approach, the results obtained in the healthy subjects and patients clearly showed that the quantified APT effect in the ischaemic tissue was smaller than in the normal tissue, consistent with the acidification theory and the reported APT effect after stroke in both preclinical and clinical studies.

As saturation was performed using Gaussian pulses with a flip angle close to 180°, it is likely that there will be some chemical exchange rotation transfer (CERT)‐related effects (present when *γB*_1_ ≥ labile proton exchange rate, where *γ* is the gyromagnetic ratio) 38. For the average power (0.55 μT) used in this study, the CERT‐related effects would be observed when the exchange rate was below 23.4 Hz. The amide proton exchange rate is expected to be 28 Hz 5 in normal tissue. When stroke occurs, the change in the measured signal at 3.5 ppm implies a slowing of the amide proton exchange rate; thus, it is likely that the CERT‐related effects would exist in the ischaemic tissue.

Finally, it should also be noted that other methods exist to quantify the APT effect, such as Lorentzian difference analysis 21 and a three offset measurement approach 34, but these methods were not considered in this study. Although they are less contaminated by effects, such as NOE, these approaches are still not able to account for all the variations discussed above. In addition, the three offset measurement technique requires the APT effect to be present as a well‐defined dip on the z‐spectra, which is not generally achievable when the acquisition is performed at 3 T.

## CONCLUSIONS

In this study, different APT quantification methods were assessed on data acquired from healthy volunteers and hyperacute stroke patients. Clear changes in the APT signal in patients within the region of ischaemia, as identified from DWI, were observed using all the quantification methods. It was found that the model‐based approach was more robust and performed better at quantifying the APT effect than the widely used asymmetry measures, such as MTR_asym_, on both the healthy volunteer and patient APT data for the acquisition settings used. Results from the model‐based analysis were employed to generate quantitative pH maps using published calibration data; relatively homogeneous pH maps were obtained in healthy subjects, and lower pH values were seen in the ischaemic tissue of patients with hyperacute stroke.

## Supporting information

Supporting info item
