# Supplementary figures and images for: Comparing different analysis methods for quantifying the MRI amide proton transfer (APT) effect in hyperacute stroke patients
Source: NMR Biomed. 2014 Jun 10;27(9):1019–29. doi: 10.1002/nbm.3147 (PMC4737232; doi:10.1002/nbm.3147)

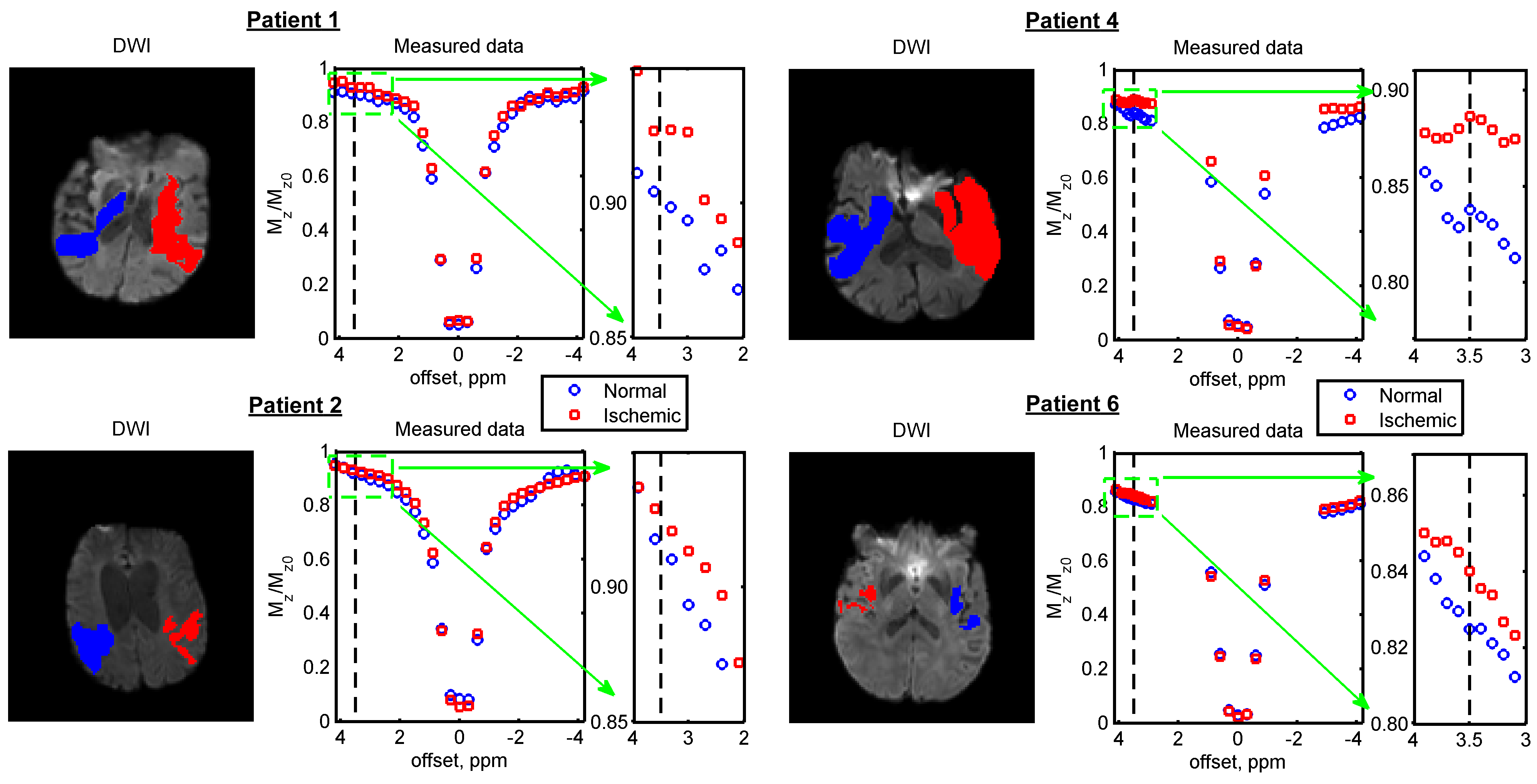

Supplement: Supplementary file 1 — Supporting info item [file NBM-27-1019-s001.tif]
